# Linking Traffic Noise, Noise Annoyance and Life Satisfaction: A Case Study

**DOI:** 10.3390/ijerph10051895

**Published:** 2013-05-07

**Authors:** Jan Urban, Vojtěch Máca

**Affiliations:** Charles University Environment Center, José Martího 2, 160 00 Praha 6, Czech Republic; E-Mail: vojtech.maca@czp.cuni.cz

**Keywords:** traffic noise, noise annoyance, life satisfaction, residential satisfaction

## Abstract

The primary purpose of this study was to explore the link between rail and road traffic noise and overall life satisfaction. While the negative relationship between residential satisfaction and traffic noise is relatively well-established, much less is known about the effect of traffic noise on overall life satisfaction. Based on results of previous studies, we propose a model that links objective noise levels, noise sensitivity, noise annoyance, residential satisfaction and life satisfaction. Since it is not clear whether a bottom-up or top-down relationship between residential satisfaction and life satisfaction holds, we specify models that incorporate both of these theoretical propositions. Empirical models are tested using structural equation modeling and data from a survey among residents of areas with high levels of road traffic noise (n1 = 354) and rail traffic noise (n2 = 228). We find that traffic noise has a negative effect on residential satisfaction, but no significant direct or indirect effects on overall life satisfaction. Noise annoyance due to road and rail traffic noise has strong negative effect on residential satisfaction rather than on overall life satisfaction. These results are very similar for the road and railway traffic contexts and regardless of whether the model assumes the top-down or bottom-up direction of the causation between life satisfaction and residential satisfaction.

## 1. Introduction

Environmental noise, and persistent high levels of transportation noise in particular, have been shown to have considerable effects on human health and well-being [1,2]. According to the WHO, one third of the EU citizens are annoyed by environmental noise and about 25% of the EU citizens experience sleep disturbances due to environmental noise [1]. According to the first round of noise mapping in agglomerations and on major roads, around 56 million people within agglomerations and 34 million people outside agglomerations across the EU are exposed to noise levels above L_den_ 55 dB [3], a limit level that has been suggested by the WHO to protect the majority of people from being seriously annoyed [4]. Railway noise seems to be a less serious problem than road traffic noise as only 6.3 million citizens in agglomerations and 5.4 million along major railways outside agglomerations in the EU are exposed to noise levels exceeding L_den_ of 55 dB, according to the first noise mapping [3]. Indeed, exposure to the same level of railway noise is likely to result in less annoyance in comparison to road traffic and aircraft noise, as documented by exposure-response functions based on a meta-analysis of empirical studies on noise annoyance [2,5], possibly with the exception of high-speed trains in Korea and Japan, which are reported to produce more noise annoyance at the same noise levels [6,7].

Despite this type of evidence, relatively little is known about how road and rail traffic noise affect overall life satisfaction. The purpose of this paper is to fill this gap by linking different concepts that have appeared in the empirical literature, proposing a unified model and submitting this model to an empirical test. Specifically, this paper aims to link noise exposure, noise sensitivity, noise annoyance, residential satisfaction and overall life satisfaction.

### 1.1. Noise Annoyance and Noise Sensitivity

Environmental noise is defined as unwanted sound caused by emissions from traffic and industrial and recreational infrastructures, which may cause annoyance and health damage. The noise originating from transport is a classic nuisance, or externality in economic terms. This is a spill-over effect of an activity which affects the welfare of others, without being compensated by the producer of the externality. One of the effects of noise exposure on the well-being of humans consists in noise annoyance defined as “a feeling of displeasure associated with any agent or condition, known or believed by an individual or group to adversely affect them” [8]. According to Miedema, noise annoyance is “a sensitive indicator of adverse noise effects and by itself means that noise affects people’s quality of life” [9]. Thus, noise annoyance appears to mediate some of the health effects of noise exposure [10].

Over the last 40 years, a substantial body of literature has provided evidence on the relationship between the levels of noise exposure and expected noise annoyance. In a seminal synthesis, Schultz [11] translated exposure-response relationships from several social surveys into common day-night average sound levels and proposed the average of those relationships as a means for predicting community annoyance from transportation noise. Miedema and Oudshoorn [5] later developed a more elaborated model for predicting three levels of noise annoyance for road, rail and aircraft noise for two alternative noise metrics: the day-night levels (mostly used in the USA) and the day-evening-night levels endorsed in the EU’s Environmental Noise Directive. In a recent study for the Danish Ministry of Science, Technology and Innovation, Pedersen *et al*. [12] developed logistic functions for exposure-response annoyance relationships with various covariates representing the effects and parameters of noise sources, locations, activities, perceived acoustic attributes and non-acoustic factors. These noise exposure functions, which describe the relationship between noise exposure and noise annoyance in probabilistic terms, typically consider noise exposure values between 45 dB and 75 dB in order to avoid uncertainty.

Noise annoyance is currently one of the most extensively studied metrics for assessment of environmental noise impacts on people. The ISO standard 15666:2003 provides a five-point scale of annoyance (not annoyed, slightly, moderately, very and extremely annoyed) for socio-acoustic and social surveys on noise effects.

Noise annoyance is influenced by many factors besides noise exposure, including person-related variables (age, stress level, duration of exposure to noise, noise sensitivity), house-related variables (floor number, number of windows oriented towards the noise source), and the characteristics of the noise source (traffic flow during the day and night) [13]. Noise sensitivity is probably the most important non-acoustic factor of noise annoyance [13,14,15,16,17,18,19,20], while socio-demographic factors usually play a minor role [21]. As a matter of fact, noise sensitivity seems to be related to an individual’s psychological characteristics, independent of noise exposure [21].

### 1.2. Life Satisfaction

Happiness, subjective well-being, and life satisfaction are some of the terms that appear interchangeably in the literature to denote the evaluation of one’s life as a whole [22,23] or specific aspects of life [24]. The concept of “overall” life satisfaction on which we focus in this paper originated in psychology and sociology, but has recently also made its way into economics, where it is used as a measure of “experienced utility” [25] and applied in a number of empirical studies that aim at monetary valuation of non-market goods [26].

Since “overall” life satisfaction is an evaluation of one’s life as a whole, it can essentially be influenced by any number of possible factors. Empirical studies show that many economic variables such as income, unemployment, and inflation are predictive of life satisfaction [22,27,28,29] as are factors that characterize one’s socio-demographic situation (age, gender, parenthood) and health status [30].

Recent research has shown that besides the socio-demographic and economic variables, ambient environmental quality, too, affects life satisfaction. It has been found that climatic conditions affect life satisfaction at the aggregated country level [31] and also at the individual level [32,33]. Similarly, air pollution has been found to affect life satisfaction at the aggregated country level [34,35,36], as well as the individual level [37,38]; one study known to us has also found an effect of perceived air pollution on life satisfaction [30]. It is important to note that factors of life satisfaction may interact in a way that either attenuates or amplifies the direct effects of those factors on life satisfaction. One example of such an interaction would be the difference in the level of life satisfaction between urban and rural areas, which is a function of the country’s economic development [39] and leads urban populations, especially in less developed countries, to express higher life satisfaction despite the serious problems that they are facing in their urban environments.

Only a few studies have examined the effect of traffic noise exposure on life satisfaction. Lercher and Kofler [40] have found a significant negative effect of traffic noise (from road and rail traffic) on overall life quality in alpine rural areas. Their finding suggests that the effect takes on a concave function and increases particularly at noise levels higher than 60 dB(A). A study by van Praag and Baarsma [41] has examined and found an effect of exposure to aircraft noise on individual life satisfaction. Another study has examined the effect of perceived adverse noise-related effects (perceived air pollution and perceived noise pollution regardless of their source) on life satisfaction [30], but has found this effect only for some of the model specifications, which may lead one to infer that the effect is not particularly strong.

Besides the studies into the effect of noise on overall life satisfaction, there are several empirical papers that focus on the effect of noise exposure and noise annoyance on satisfaction with some aspects of one’s life (as opposed to satisfaction with one’s life as a whole). Probably the most studied aspect of life satisfaction found to be related to noise is residential satisfaction or one’s satisfaction with the quality of life in a specific area. A study by Botteldooren *et al*. [42] has found an effect of road traffic noise exposure on residential quality. A study by Schreckenberg *et al*. [21] has examined the effects of exposure to aircraft noise and related noise annoyance on different aspects of quality of life (*i.e*., satisfaction with dwelling, residential area, infrastructure, quietness, and attractiveness of the residential area) and found that there was a significant negative effect of noise exposure on the attractiveness of the residential area and the total score of residential satisfaction, and also a significant effect of noise annoyance on satisfaction with dwelling. A study by Kroesen *et al*. [43] has found an effect of exposure to aircraft noise on residential satisfaction mediated by noise annoyance.

There is also additional evidence concerning the effect of traffic noise on different aspects of human well-being that comes from studies of health-related quality of life. For instance, a study by Shepherd *et al*. [44] has found the effect of aircraft noise, mediated by annoyance, on quality of life (measured as a multidimensional construct that included physical health, psychological well-being, social relationships and environmental factors). A study by Dratva *et al*. [45] has found an effect of road traffic noise on all aspects of health-related quality of life (physical functioning, physical role, bodily pain, vitality, social functioning, emotional role, and mental health) except general health. However, the (overall) life satisfaction used in this study is conceptually different from health-related quality of life [46] and effects of traffic noise on health-related quality of life therefore do not necessarily imply that (overall) life satisfaction shall be affected too.

As far as we are aware, the direction of the causation between overall life satisfaction and residential satisfaction has not been established by previous research. This is closely related to a more general debate in the life satisfaction literature between proponents of so-called “bottom-up theories” of subjective well-being, who argue that domain satisfactions affect overall life satisfaction, and proponents of “top-down theories”, who propose that the causation runs in the opposite direction [47]. Apparently, there is no general solution to the top-down *vs.* bottom-up debate because the direction of the causation is specific to each domain satisfaction and, in addition, can be bidirectional, spurious, or any combination of these [48], and can even be unstable across models and datasets [49].

Studies known to us assume that residential satisfaction affects life satisfaction in a bottom-up fashion (see, e.g., [50,51]). However, no empirical study has specifically examined the direction of the causation between residential satisfaction and life satisfaction and this question therefore remains open.

The cross-sectional evidence exploited in this study is not particularly suitable to testing alternative hypotheses about the direction of the causation. In fact, we can detect whether one or the other direction of the causation assumed in the model leads to a higher or lower misfit of the model but there is no way to compare the misfit of the two alternative models statistically because these are non-nested models.

Assuming bottom-up causation or top-down causation between life satisfaction and residential satisfaction can lead to different results and their interpretations regarding the effect of noise on life satisfaction. For this reason, and because we are unable to establish the direction of the causation between residential satisfaction and life satisfaction, we propose and empirically test models that specify either a bottom-up or a top-down relationship between the two variables.

## 2. Data and Method

### 2.1. Model

Based on a review of previous empirical research, we propose two models that differ only in whether they assume a top-down (Figure 4, models C and D) or a bottom-up (Figure 4, models A and B) relationship between overall life satisfaction and residential satisfaction. In all the models, we assume that noise exposure has an effect on noise annoyance. This hypothesis is supported by the bulk of the noise annoyance literature discussed earlier. We also assume that noise exposure may have a direct effect on life satisfaction that is not mediated by noise annoyance. This hypothesis reflects the fact that noise annoyance is only one of several adverse effects on human well-being, albeit one of the most important [1]. We do not assume that traffic noise affects noise sensitivity because no such effect has been found by previous research [21] and we treat the two variables as exogenous.

Furthermore, we assume that noise annoyance has an effect on residential satisfaction. This assumption is supported by a wealth of evidence from previous empirical studies [21,42,43,52]. In addition, we assume that noise annoyance has a direct effect on life satisfaction unmediated by residential satisfaction [30,40,41].

As mentioned in Section 1.2, the direction of the causation between residential satisfaction and life satisfaction has not been established and for that reason we test a model that assumes a bottom-up effect of residential satisfaction on life satisfaction (path diagrams A and B in Figure 4) and also a model that assumes a top-down effect of life satisfaction on residential satisfaction (path diagrams C and D in Figure 4).

### 2.2. Measures

The noise data are taken from strategic noise maps drawn up pursuant to the EU Environmental Noise Directive and refer to the base year 2006. We make no adjustments to reflect changes that may have taken place in the meantime, such as change in traffic, installation of noise barriers, laying of silent pavements, introduction of new speed limits, *etc*., because information concerning these changes is very scarce. The fact that three years have passed between the time of measurement of objective noise levels and the time of our survey may bias the results and we acknowledge this limitation. Unfortunately, no newer data of comparable quality are available and will only be available after the completion of the 2012 strategic noise mapping. Nonetheless, as we argue in the concluding section of this paper, even with this limitation we think that the data capture the overall trend sufficiently.

To capture noise exposure, we have opted for the 24-hour composite noise indicator *L_den_* that is nowadays a common reference of the objective noise level used in socio-acoustic research [5,9] and as such provided in the strategic noise maps.

To measure noise annoyance, respondents were given the following question: “When you are at home, are you annoyed by road traffic/railway noise?” (the source of noise was chosen depending on the principal noise burden). A five-point annoyance scale defined by the ISO standard 15666:2003, with the answer categories “not annoyed”, “slightly annoyed”, “moderately annoyed”, and “very and extremely annoyed” was then used to record the respondents’ annoyance.

Although various measures of life satisfaction exist, it has been shown that this concept has a high degree of consistency, reliability, validity and stability over time [47]. Indeed, it appears that different measures of life satisfaction do in fact converge and they seem to represent a single concept; they correlate with physical manifestations of happiness and people who express high life satisfaction are also more likely to be described as happy by other people and are less likely to commit suicide [35].

In the present research, we adopt a measure of life satisfaction proposed by Cantril [53], which asks people how satisfied they are with their life as a whole. This question accompanied with an 11-point scale has become almost the standard in life-satisfaction research and has been applied in a number of empirical studies [30,33,38,54,55] although some researchers may prefer to use a shortened answer scale (*cf.* [31,35]). The validity of this particular measure of life satisfaction has been demonstrated [22,23].

There seems to be no agreement whether life satisfaction should be treated as a cardinal or ordinal measure, though it appears that in many applications accepting the assumption of cardinality or ordinality will have a negligible effect on the empirical results [56]. To stay on the safe side, we decided to treat the variable as ordinal.

Residential satisfaction is measured by asking respondents the following question: “All things considered, how satisfied are you with the life in the area where you are currently living? (Take into account the area within walking distance of your home.)”. Respondents indicated their answers on an 11-point Likert-type scale ranging from 0 (“extremely dissatisfied”) to 10 (“extremely satisfied”).

Noise sensitivity is measured as the respondents’ perception of their sensitivity to noise. Respondents were asked the following question: “To what degree are you, in your opinion, sensitive to noise?” Respondents indicated their answers on a four-point Likert-type scale ranging from 1 (“very sensitive”) to 4 (“not sensitive”). We have collapsed categories 1 and 2 and categories 3 and 4 in order to make noise sensitivity a dummy variable. This procedure allows us to include noise sensitivity as an observed dummy variable in the models depicted in Figure 4 below. Note that the model estimates were practically the same whether we included noise sensitivity as a dummy variable, a variable with four categories (assuming cardinality of the measure) or three dummy variables (simple contrast coding).

### 2.3. Data

**Figure 1:**
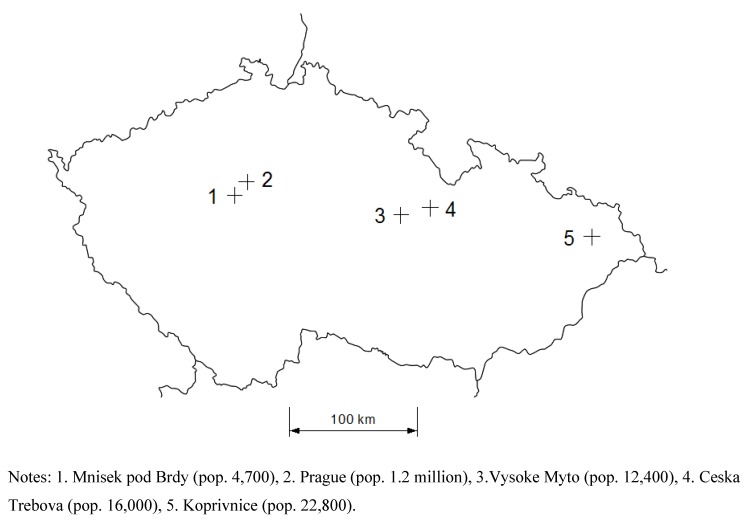
Cities included in the survey.

The data exploited in this paper come from a survey conducted in the summer of 2009 in five cities in the Czech Republic (Prague, Vysoke Myto, Ceska Trebova, Mnisek pod Brdy, and Koprivnice) using a combination of purposive and stratified random sampling. The five cities were chosen to represent the rail and road transport modes, places of different sizes and with different transport networks (*cf.* Figure 1).

Based on the noise maps, obtained from the National Reference Centre for Community Noise, the Transport Research Centre and the company Akustika Praha, we have chosen several sites in each of the cities exposed either to road traffic noise or rail traffic noise, but not both. On each of these sites, a random sample of address points was drawn. At each of these addresses, interviewers chose randomly the floor of the building, the housing unit and the respondent within that housing unit using the Kish sampling method [57]. Since we know the address points, we can estimate the noise level to which each resident has been exposed.

The data were collected using in-person interviews at the respondents’ homes. A total of 609 questionnaires were collected, out of which 363 were in areas exposed to road traffic noise and 246 in areas exposed to railway noise. However, for nine respondents exposed to road noise (all but one in Prague) and 18 respondents exposed to railway noise (all in Prague), the interviewers failed to report their precise address and therefore we could not attribute any noise levels to these respondents. As a consequence, we included 354 respondents exposed to road traffic noise and 228 respondents exposed to railway noise in the final analysis.

The average age of the respondents in our sample was 47.4 years (min. 18, max. 88) and did not differ between the cities (F = 0.515, df = 4, *p* = 0.72) or the type of noise burden, *i.e.*, road *vs.* railway (F = 0.525, df = 1, *p* = 0.52). On average, 6.7% of our respondents had only elementary education, 30.3% had high-school education without the state leaving exam, 42.1% had high-school education with the state leaving exam, 4.93% had completed college education, and 16.0% had received a university degree; this proportion is similar across the two types of noise burdens (χ^2^ = 1.956, df = 5, *p* = 0.855) but not across the five cities (χ^2^ = 47.6, df = 20, *p* < 0.001). A close look at the contingency table (not reported here to save space) reveals that the respondents in Prague were more often university-educated and less likely to have elementary education only, and that respondents in Koprivnice were less likely to have a university degree. These irregularities are due to the fact that Prague is large city that hosts several universities and attracts university-educated people, while Koprivnice is a town with a population of 22,800 that was once an important car-manufacturing center (Tatra Koprivnice) and still relies substantially on industrial production. Males represented 58.1% of our sample and their proportion was similar across the two types of noise burden (χ^2^ = 0.7, df = 1, *p* = 0.4) and across the five cities (χ^2^ = 4.81, df = 4, *p* = 0.31).

### 2.4. Procedure

The empirical models are tested using structural equation modeling [58]. SEM is very advantageous for the purpose of our study specifically because it allows us to examine a relatively complex model which includes the observed continuous variables *Noise′* and *Noise sensitivity′*, and three latent variables indicated by ordinal outcome variables. Incidentally, SEM has been previously used with some success to analyze the link between noise exposure, noise annoyance and adverse psycho-physical effects of noise exposure [14].

The core idea of SEM is that it is possible to reproduce a population variance-covariance matrix if we know the model that correctly explains the variation in the data. Although different estimators may be used to estimate the model parameters, all the estimators basically aim to minimize the discrepancy between the model-implied and empirical variance-covariance matrix of the observed variables.

Our model could be expressed as a series of three equations for each of the dependent variables. In the shortened matrix form, these equations can be re-expressed for each individual, *i*, as:

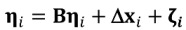
(1)
where **η** (3 × 1) is a vector of the endogenous latent variables (*i.e.*, *Noise annoyance*, *Residential satisfaction*, and *Life satisfaction*), **x** (2 × 1) is a vector of the observed exogenous variables (*Noise′* and *Noise sensitivity′*), **B** (3 × 3) is a matrix of the regression coefficients among **η**’s with the diagonal elements equal to zero and **I-B**, being a non-singular matrix, **Δ **(3 × 2) is a parameter vector of the coefficients for the regression of the latent variables *η*’s on manifest variables *x*’s, and **ζ **(4 × 1) is a vector of the residuals that are assumed to have a multivariate normal distribution. Note that several elements in the **Δ **matrix are fixed to zero because we assume no direct effect of *Noise sensitivity′* on *Residential satisfaction* and *Life satisfaction* and also no direct effect of *Noise′* on *Residential satisfaction* (see also Figure 4).

We assume that the *j*-th continuous latent variable, *η_j_*, is measured perfectly (without any measurement error) by only one observed continuous outcome variable, *y*, expressed as the *i*-th respondent’s rating on the *j*-th Likert-type scale. The probability of observing the *i*-th respondent indicating values 0,1,...,S on this scale can be expressed using the following ordered probit model (for technical details of this model see, e.g., [59]):

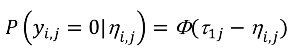
(2)

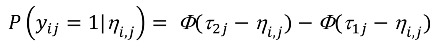
(3)

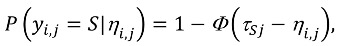
(4)
where Φ is the standardized univariate normal distribution function and τ’s are the estimated threshold parameters.

Notice that the variables *Noise annoyance*, *Residential satisfaction*, and *Life satisfaction* are truly latent variables, although they are each measured by one observed indicator only. These observed indicators, denoted by the prime sign (′) as *Noise annoyance′*, *Residential satisfaction′* and *Life satisfaction′*, are ordinal variables linked to their respective underlying latent variables *Noise annoyance*, *Residential satisfaction*, and *Life satisfaction*, through a threshold model given in Equations (2–4), which is essentially an ordinal probit model. On the other hand, the observed traffic noise captured by the variable *Noise′* and the self-reported *Noise sensitivity′* enter the structural part of the model given in Equation (1) directly because *Noise′* is a continuous variable and *Noise sensitivity′* is a dummy variable. This distinction between the latent variables (albeit ones measured by only one observed indicator) and the observed variables is important because the estimated path coefficients (reported in Figure 4) relate to the relationships between the latent variables *Noise annoyance*, *Residential satisfaction*, *Life satisfaction* and not their manifest indicators. We maintain this distinction between the latent variables and their observed indicators throughout the paper (*cf.* Table 3 and Figure 4).

To estimate the model parameters, we use limited-information estimation with a robust weighted least-square estimator which avoids demanding numerical integration in the ML estimation, and which is also robust in the presence of non-normal outcome variables [60]. The models are estimated in the MPlus software, version 6.1 [61].

## 3. Results

**Figure 2:**
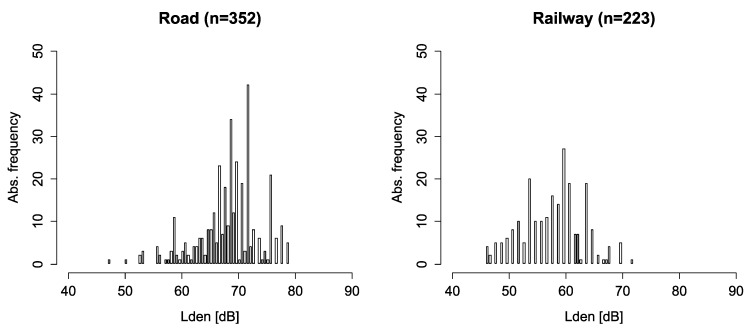
Sample noise exposure (*Noise*)—Road traffic and railway traffic.

The sample exposure to traffic noise in terms of L_den_ ranged between 47 and 79 dB for road traffic noise and between 46 and 72 dB for rail traffic noise. Figure 2 shows the distributions of noise exposure in the samples exposed to road traffic noise and rail traffic noise.

The average noise exposure for the two samples is further examined separately for the cities from which the samples were drawn in Table 1. The mean level of traffic noise exposure was higher in the road traffic sample (*t* = 22.04, df = 576.9, *p* < 0.001). The results of the ANOVA tests conducted separately for the road traffic and rail traffic samples suggest that there were significant differences in exposure to noise levels among the cities.

Table 2 shows that the average noise annoyance levels in the sub-samples coming from different cities are quite high with mean values of 4.13 and 3.45 for road and rail traffic annoyance respectively (where 1 stands for “not annoyed” and 5 for “extremely annoyed”). The average noise annoyance in the road traffic sub-sample is higher than that in the rail traffic sub-sample (*t* = 7.55, df = 431.1, *p* < 0.001). The level of annoyance is not statistically different among the sites sampled for road traffic annoyance and also among the sites sampled for rail traffic noise.

**Table 1:** Noise exposure (L_den_) in samples from different cities (means, standard deviations and ANOVA).

City	Road traffic			Railway traffic		
	N	M	SD	N	M	SD
Prague	210	70.96	4.35	117	59.97	3.81
Vysoke Myto	39	63.34	6.27	-	-	-
Mnisek pod Brdy	28	64.47	4.15	-	-	-
Koprivnice	77	65.79	4.37	-	-	-
Ceska Trebova	-	-	-	111	56.04	6.14
Total	354	68.48	5.50	228	58.06	4.45
ANOVA	F = 52.76(3), *p* < 0.001			F = 34.285(1), *p* < 0.001		

**Table 2:** Average noise annoyance scores in sub-samples from different cities (means, standard deviations and ANOVA test of equality).

City	Road traffic			Rail traffic		
	N	M	SD	N	M	SD
Prague	210	4.07	0.91	117	3.50	1.22
Vysoke Myto	39	4.08	1.04	-	-	-
Mnisek pod Brdy	28	4.57	0.92	-	-	-
Koprivnice	77	4.14	0.93	-	-	-
Ceska Trebova	-	-	-	111	3.36	1.25
Total	354	4.13	0.93	246	3.43	1.23
ANOVA	F = 2.440(3), *p* = 0.064			F = 0.684(1), *p* = 0.409		

Table 3 below displays the mean levels of life satisfaction in the sub-samples coming from different cities and separately for the road traffic and rail traffic sub-samples. The average life satisfaction is lower in the road traffic sub-sample (*t* = −4.43, df = 580, *p* < 0.001). The results of the ANOVA test suggest that the mean life satisfaction level is not equal in the four cities sampled for road traffic noise but they are equal in the two cities sampled for rail traffic noise. Table 3 also displays city-specific correlations between life satisfaction and traffic noise and noise annoyance together with their significance levels. All the city-specific correlations between traffic noise levels and life satisfaction are low and statistically insignificant. The city-specific correlations between noise annoyance and life satisfaction are generally insignificant with the exception of the negative and statistically significant correlation between noise annoyance and life satisfaction that can be observed in the road traffic sub-sample from Prague. Nonetheless, the statistical test of equality of the correlation coefficients based on Fischer’s z-transformation of the sample correlation coefficients does not reject the null of equality of city-specific correlation coefficients between life satisfaction, noise annoyance and traffic noise within the road and the rail sub-samples.

**Table 3:** Life satisfaction in sub-samples from different cities.

Town	Road traffic					Rail traffic				
	Life satisfaction			Correlation of LS with		Life satisfaction			Correlation of LS with	
	N	M	SD	Noise	Noise annoyance	N	M	SD	Noise	Noise annoyance
Prague	210	7.39	2.247	0.029	−0.133 *	135	8.04	2.397	−0.07	0.007
Vysoke Myto	39	7.78	1.874	0.168	−0.138	-	-	-	-	-
Mnisek pod Brdy	28	7.25	2.504	−0.254	−0.096	-	-	-	-	-
Koprivnice	77	6.12	2.071	0.029	−0.18	-	-	-	-	-
Ceska Trebova	-	-	-	-	-	111	7.95	2.125	−0.059	0.034
Total	354	7.15	2.25	0.066	−0.140 **	228	8.00	2.27	−0.065	0.020
Tests of equality	^†^F = 7.694(4), *p* < 0.001			^‡^χ^2^ = 0.185(3), *p* = 0.97	^‡^χ^2^ = 2.78(3) *p*=0.43	^†^F = −0.094(1), *p* = 0.76			^‡^χ^2^ = 0.007(1), *p* = 0.93	^‡^χ^2^ = 0.043(1), *p* = 0.84

*Notes*: * *p* < 0.05; ** *p* < 0.01. **^†^** ANOVA. **^‡^** Statistical test of equality of correlation coefficients based on Fischer’s z-transformation of the sample correlation coefficients.

**Figure 3:**
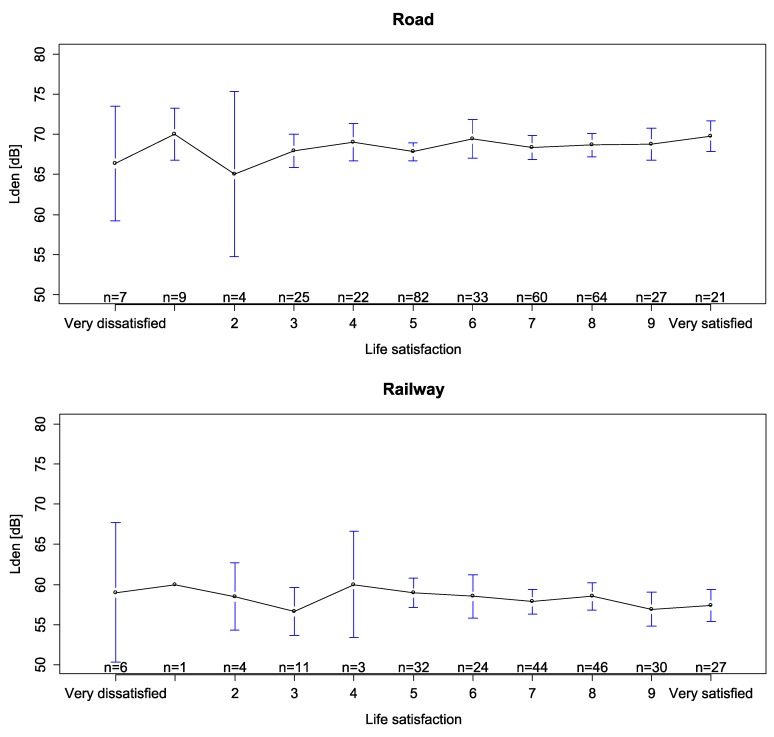
Average noise exposure for each of the life satisfaction levels (mean and its 95% conf. int.).

Figure 3 displays the average noise exposures for different levels of life satisfaction separately for the samples exposed to road and rail traffic noise. At first sight, there appears to be hardly any clear monotonous relationship, especially when we take into account the estimated confidence intervals of average noise exposure for each level of life satisfaction.

The zero-order correlations between the observed variables are reported in Table 4. The correlations between noise exposure and life satisfaction equal 0.066 and −0.065 for the road and rail traffic sub-samples, respectively, and are not statistically significant at the 5% level. The correlations between noise annoyance and life satisfaction are −0.140 (significant at the 1% level) for road traffic and 0.020 (statistically insignificant at the 5% level) for rail traffic noise.

**Figure 4:**
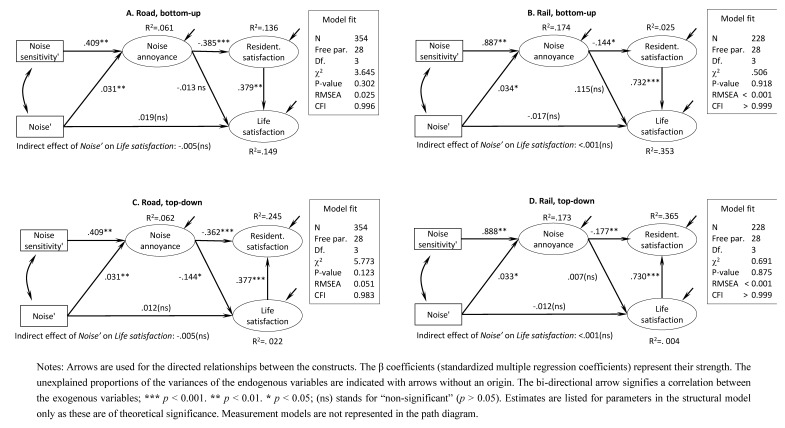
Model estimates.

The estimates of the models are displayed in Figure 4 for road traffic noise (models A and C) and rail traffic noise (models B and D), with bottom-up (models A and B) and top-down (models C and D) specifications of the relationship between life satisfaction and residential satisfaction.

The fit of models A, B and D is excellent (χ^2^(3) < 3.6; RMSEA < 0.025; CFI > 0.99). The fit of model C is somewhat worse (χ^2^(3) < 5.8; RMSEA < 0.051; CFI > 0.98) but still relatively good as the CFI is above the threshold of 0.9 suggested for well-fitting models [62], the RMSEA is very close to the cut-off value of 0.05 recommended for well-fitting models and certainly well below the threshold of 0.08 suggested as an indication of acceptable fit [63], and the relative χ^2^ (ratio of χ^2 ^to degrees of freedom) is below the value of 2 suggested as an indication of good fit [64]. The fit indices do not suggest that either a bottom-up or a top-down specification would lead to poorly fitting models. However, direct comparison of the fit of the two model specifications is hindered by the fact that they are non-nested and therefore statistical comparison of their fit indices based on the χ^2 ^values is not possible. For these reasons, we consider the results from all the four models.

**Table 4:** Means, standard deviations, and correlations between observed variables.

ROAD	M	SD	1	2	3	4	5	M	SD	RAIL
1.* Noise ′*	68.48	5.50	1	0.549 **	*0.020*	*−0.064*	*−0.065*	*8.00*	*2.27*	1. *Life satisfaction ′*
2.* Noise sensitivity ′*	2.12	0.69	−0.105 *	1	−0.141 *	*−0.011*	*−0.058*	*7.84*	*2.17*	2. *Residential satisfaction ′*
3.* Noise annoyance ′*	4.12	0.93	0.142 **	−0.169 **	1	−0.381 **	0.172 **	*3.45*	*1.22*	3. *Noise annoyance ′*
4.* Residential satisfaction ′*	6.44	2.59	−0.132 *	0.029	−0.328 **	1	*−0.095*	*2.20*	*0.84*	4. *Noise sensitivity ′*
5.* Life satisfaction ′*	7.15	2.25	0.066	0.068	−0.140 **	0.370 **	1	*58.06*	*5.44*	5. *Noise ′*

*Notes*: Figures in Roman type in the lower triangle relate to the road traffic noise sub-sample; figures in italics in the upper triangle relate to the rail traffic sub-sample. * *p* < 0.05. ** *p* < 0.01.

Some of the results presented in Figure 4 are common to all the four models, regardless of the source of traffic noise (rail *vs*. road) and model specification (top-down *vs*. bottom-up): (1) noise annoyance is positively and statistically significantly related to both noise and noise sensitivity; (2) noise annoyance has a negative effect on residential satisfaction; (3) residential satisfaction and life satisfaction are positively related, and (4) noise annoyance has a negative effect on residential satisfaction. Indeed, all these findings are in line with the findings of previous empirical studies.

Regarding the effect of noise on life satisfaction, the results presented in Figure 4 suggest that noise has no statistically significant direct or indirect (*i.e*., mediated by noise annoyance and residential satisfaction) effect on life satisfaction regardless of the source of traffic noise or model specification. Noise annoyance appears to have a weak negative but still statistically significant effect on life satisfaction only in the top-down model for rail traffic noise.

The proportion of the explained variance of residential satisfaction and life satisfaction depends on whether a top-down or a bottom-up specification of the model is assumed. The proportion of the explained variance of noise annoyance is about 6% and 17% for the road and rail traffic sub-samples, respectively.

Table 5 reveals the pairwise tests of equality of the regression coefficients recommended by Paternoster *et al*. [65]. Using this test, we can determine whether the parameter estimates are different among the four models. Note that since bottom-up and top-down models differ in the direction of the causation that they assume between residential satisfaction and life satisfaction, it is meaningful to compare the estimates of path coefficients between these two specific variables only within the groups of bottom-up or top-down models but not between them.

**Table 5:** Pairwise comparison of structural parameters in the 4 models.

Effect	Test of equality of structural parameters *					
	Rail bu = road bu	Rail bu = rail td	Rail bu = road td	Road bu = rail td	Road bu = road td	Road td = rail td
*Life satisfaction* regressed on						
Noise′	−1.854	−0.243	−1.494	1.820	0.450	−1.409
Noise annoyance	1.413	1.283	**3.027**	−0.226	1.457	1.809
Residential satisfaction	**4.066**		-	-	-	-
*Residential satisfaction* regressed on						
Noise annoyance	2.727	**−9.125**	**−6.697**	**−11.563**	**−9.695**	**2.013**
*Noise annoyance* regressed on						
Noise* ′*	0.176	0.054	0.176	−0.117	<0.001	0.117
Noise sensitivity *′*	**−2.249**	0.004	**−2.249**	**2.254**	<0.001	**−2.254**

Notes: * The test statistics have a standard normal distribution. Bold type indicates values of the test statistics which exceed the value of 1.96, implying rejection of H0 (equality of coefficients) at the 5% level of statistical significance; “bu” stands for bottom-up and “td” stands for top-down model specifications.

The pairwise equality tests displayed in Table 5 suggest that the path coefficients for the effect of noise on life satisfaction are not statistically different in the four models. The effect of noise on noise annoyance is also not statistically different among the four models. The effect of noise annoyance on life satisfaction is different only between the rail bottom-up and road top-down models. The effect of noise annoyance on residential satisfaction is quite variable and differs in five out of the six pairs of models. The effect of life satisfaction in the top-down specification is different for road and railway noise. Finally, also the effect of noise sensitivity on noise annoyance appears to be quite variable as it is significantly different in four out of the total of six pairs of models.

## 4. Discussion

This study aimed to answer the question whether road and rail traffic noise negatively affects overall life satisfaction. Our results suggest that the level of traffic noise from road and rail traffic has no statistically significant direct or indirect effect on life satisfaction in the five studied Czech cities and specifically on life satisfaction of the residents in areas exposed to higher levels of traffic noise. We only find a weak, yet statistically significant effect of noise annoyance on life satisfaction in one of the four empirical models estimated. Other than that, our study confirms several findings of previous studies, namely the positive effect of noise sensitivity and noise exposure on noise annoyance, the negative effect of noise annoyance on residential satisfaction, and the strong association between residential satisfaction and life satisfaction.

As a matter of fact, only three studies have addressed the effect of traffic noise on life satisfaction up to now and they all have found some effect of traffic noise on life satisfaction. The study by Lercher and Kofler [40] has found a significant negative effect of traffic noise (from road and rail traffic) on overall life quality in alpine rural areas. Their findings suggest that the effect takes on a concave function and increases particularly at noise levels higher than 60 dB(A). We would argue, however, that the specific topographic features of the study (alpine valleys), the rural context with low background noise, and the specific type of transport (heavy truck loads during the night) might have strengthened the link between traffic noise and life satisfaction observed in the study.

In a similar vein, the effect of aircraft noise on life satisfaction observed in the study by van Praag and Baarsma [41] can also be expected to be much stronger than what we observe in our study due to the exceptionally high noise levels and the particular features of aircraft noise. Finally, the study by Rehdanz and Maddison [30], which examined the effect of perceived noise-related adverse effects (perceived air pollution and perceived noise pollution regardless of their source) on life satisfaction [30], found this effect only for some of the model specifications, which may lead one to infer that the effect is not particularly strong.

We think that the lack of an effect of noise on life satisfaction in our data is due to a combination of three aspects of the present study: its focus on the urban environment, its focus on rail and road traffic noise, and its focus on overall life satisfaction. Arguably, the effect of traffic noise may be much stronger in rural areas [40] and for aircraft noise, especially at higher levels [41]. Indeed, as previous studies have shown, some aspects of life satisfaction such as residential satisfaction are negatively influenced by road traffic noise [43] and aircraft noise [21,43] for that matter. The present study confirms this contention as we find a negative effect of road and rail traffic noise annoyance on residential satisfaction, while the effect of noise annoyance on overall life satisfaction is weaker and statistically significant in only one of the four models estimated.

In the remainder of this section, we shall address some of the concerns about the results presented in this paper.

Although all the models estimated in this study fit to empirical data quite well and the estimates of the model parameters are generally in line with what we can learn from other studies, a closer look at the path diagrams depicted in Figure 4 reveals that all the models leave a sizable proportion of the variance in the dependent variables unexplained. This result is not surprising if we take into account the complexity of phenomena such as noise perception and life satisfaction and the relative simplicity of our model. As a matter of fact, noise annoyance is a product of such diverse factors as person-related variables (age, years of employment, stress score, duration of stay at the accommodation during the day), house-related variables (living room and/or bedroom windows oriented towards the street, the floor number), and neighborhood-related variables (L_eq_ for day and night, maximum night-time noise level, traffic flow during the day and night) [13]. Life satisfaction also appears to be a phenomenon of considerable complexity and even relatively elaborate individual-level models explain only between 4 and 9% [54], between 3 and 5% [55], between 8 and 9% [30] or about 9% of the variability in life satisfaction [38].

Another issue we should address here is that all the models estimated in this study assume noise sensitivity to be an exogenous variable, following the finding of a previous study by Schreckenberg *et al*. [21]. Our analysis (see Table 4) reveals that noise sensitivity is weakly yet statistically significantly (r = −0.105, *p* < 0.05) correlated with traffic noise in the road traffic sub-sample. Note also that the correlation between noise sensitivity and traffic noise in the rail traffic sub-sample is of a similar magnitude (r = −0.095), albeit not statistically significant (which may be due to the smaller size of the rail sub-sample). These findings are consistent with the contention that “higher moving rates in urban sites may lead to selection, leaving back the poor and the noise-resistant in the high noise level zones” [40]. Nonetheless, our data do not support this hypothesis as the partial correlation between the length of residence in the area and noise sensitivity, controlling for noise levels, is statistically significant but positive (r_partial_ = 0.153, *p* < 0.001), meaning that newcomers are less noise-sensitive than residents living in the area for a longer period of time. It is important to mention here that Czechs are rather hesitant to change their place of residence (the mean length of residence at the current place was 23 years in our sample) and, therefore, these results should not be generalized to other countries. In any case, the inclusion of noise sensitivity in our models as an endogenous variable with respect to noise does not change substantially the estimated direct and indirect effects of noise on residential satisfaction and on life satisfaction.

Another thing we may be worried about is whether the heterogeneity of the cities included in our survey compromises our results. This is particularly the case of the sub-sample of respondents from Prague, who seem to have higher education levels than respondents from the other cities (see Section 2.3). Indeed, the respondents from Prague are exposed to higher average levels of traffic noise than the respondents from the other cities (see Table 1) and yet, their life satisfaction is not considerably lower—it is actually the second highest in the road sub-sample and the highest in the rail sub-sample (see Table 3). These observations are consistent with the findings of other studies that life satisfaction tends to be higher in developed urban areas despite specific urban problems, including noise pollution (*cf.* [39]).

Although we cannot rule out the hypothesis that unobserved city-specific factors related to economic development attenuate the relationship between life satisfaction and traffic noise in our data, we think that such an explanation for the missing link between noise and life satisfaction is not sufficient. Firstly, differences in life satisfaction between developed urban areas and less developed ones should be relatively smaller in a post-transition country such as the Czech Republic [39], not mentioning the fact that we included only urbanized areas in our survey. Secondly, empirical evidence from the European Social Survey conducted in 2004 does not suggest that there are differences in average life satisfaction between urban and rural areas in the Czech Republic [66]. Thirdly, none of the city-specific correlations between life satisfaction and traffic noise is significant in our sample and they are not statistically different (see Table 3) within the road and rail traffic sub-samples.

A number of limitations of the present study should be acknowledged. Firstly, our data come from a survey of an urban population exposed to rather high levels of traffic noise. This fact by itself is likely to attenuate the correlation between noise exposure (and likely also noise annoyance) on the one hand and life satisfaction and residential satisfaction on the other simply because the variance in the noise exposure is lower than would be in a sample of general population. On the other hand, we have been able to find an effect of noise annoyance on residential satisfaction. This implies that even if the restricted variance of the noise levels attenuates the effect of either noise or noise annoyance on life satisfaction, this effect must be considerably weaker than that on residential satisfaction.

Another limitation of the present study consists in the fact that there has been a three-year gap between the collection of the noise exposure data and our survey. Our study most likely underestimates the noise exposure because the traffic has increased over these three years (especially the road traffic). However, from what we can learn about the sites that our data come from, we are not aware of any abrupt increases in the traffic flow over the past three years. For this reason, we believe that the effects of noise exposure (but not the average level of noise exposure) are not severely biased in our study.

Our study also suffers from a number of limitations that are rather generic to cross-sectional studies. Specifically, the relatively small sample sizes disadvantage our study particularly by decreasing its statistical power. This may attenuate the correlations between the variables. We also have to bear in mind that our data are cross-sectional and therefore their relevance for the testing of the causal hypothesis is lower than that of longitudinal or experimental data [14,67]. On the other hand, we firmly agree with Rubin [68] that no data can principally prove a causal hypothesis and that different types of data (experimental, observational, *etc.*) differ only in the level of their relevance for the testing of the causal hypotheses.

## 5. Conclusions

This study finds that road and rail traffic noise has no statistically significant direct or indirect effect on overall life satisfaction in urban areas. Noise annoyance due to road and rail traffic noise has a strong negative effect on residential satisfaction rather than on overall life satisfaction.
